# Effect of Household-Based Drinking Water Chlorination on Diarrhoea among Children under Five in Orissa, India: A Double-Blind Randomised Placebo-Controlled Trial

**DOI:** 10.1371/journal.pmed.1001497

**Published:** 2013-08-20

**Authors:** Sophie Boisson, Matthew Stevenson, Lily Shapiro, Vinod Kumar, Lakhwinder P. Singh, Dana Ward, Thomas Clasen

**Affiliations:** 1 Department of Disease Control, Faculty of Tropical and Infectious Diseases, London School of Hygiene & Tropical Medicine, London, United Kingdom; 2 Indian Institute of Health Management Research, Jaipur, India; 3 Population Services International, New Delhi, India; University of East Anglia, United Kingdom

## Abstract

**Background:**

Boiling, disinfecting, and filtering water within the home can improve the microbiological quality of drinking water among the hundreds of millions of people who rely on unsafe water supplies. However, the impact of these interventions on diarrhoea is unclear. Most studies using open trial designs have reported a protective effect on diarrhoea while blinded studies of household water treatment in low-income settings have found no such effect. However, none of those studies were powered to detect an impact among children under five and participants were followed-up over short periods of time. The aim of this study was to measure the effect of in-home water disinfection on diarrhoea among children under five.

**Methods and Findings:**

We conducted a double-blind randomised controlled trial between November 2010 and December 2011. The study included 2,163 households and 2,986 children under five in rural and urban communities of Orissa, India. The intervention consisted of an intensive promotion campaign and free distribution of sodium dichloroisocyanurate (NaDCC) tablets during bi-monthly households visits. An independent evaluation team visited households monthly for one year to collect health data and water samples. The primary outcome was the longitudinal prevalence of diarrhoea (3-day point prevalence) among children aged under five. Weight-for-age was also measured at each visit to assess its potential as a proxy marker for diarrhoea. Adherence was monitored each month through caregiver's reports and the presence of residual free chlorine in the child's drinking water at the time of visit. On 20% of the total household visits, children's drinking water was assayed for thermotolerant coliforms (TTC), an indicator of faecal contamination. The primary analysis was on an intention-to-treat basis. Binomial regression with a log link function and robust standard errors was used to compare prevalence of diarrhoea between arms. We used generalised estimating equations to account for clustering at the household level. The impact of the intervention on weight-for-age z scores (WAZ) was analysed using random effect linear regression.

Over the follow-up period, 84,391 child-days of observations were recorded, representing 88% of total possible child-days of observation. The longitudinal prevalence of diarrhoea among intervention children was 1.69% compared to 1.74% among controls. After adjusting for clustering within household, the prevalence ratio of the intervention to control was 0.95 (95% CI 0.79–1.13). The mean WAZ was similar among children of the intervention and control groups (−1.586 versus −1.589, respectively). Among intervention households, 51% reported their child's drinking water to be treated with the tablets at the time of visit, though only 32% of water samples tested positive for residual chlorine. Faecal contamination of drinking water was lower among intervention households than controls (geometric mean TTC count of 50 [95% CI 44–57] per 100 ml compared to 122 [95% CI 107–139] per 100 ml among controls [*p*<0.001] [*n* = 4,546]).

**Conclusions:**

Our study was designed to overcome the shortcomings of previous double-blinded trials of household water treatment in low-income settings. The sample size was larger, the follow-up period longer, both urban and rural populations were included, and adherence and water quality were monitored extensively over time. These results provide no evidence that the intervention was protective against diarrhoea. Low compliance and modest reduction in water contamination may have contributed to the lack of effect. However, our findings are consistent with other blinded studies of similar interventions and raise additional questions about the actual health impact of household water treatment under these conditions.

**Trial Registration:**

ClinicalTrials.gov NCT01202383

*Please see later in the article for the Editors' Summary*

## Abstract 2

Sophie Boisson and colleagues conducted a double-blind, randomized placebo-controlled trial in Orissa, a state in southeast India, to evaluate the effect of household water treatment in preventing diarrheal illnesses in children aged under five years of age.

*Please see later in the article for the Editors' Summary*

## Editors' Summary

**Background:**

Millennium Development Goal 7 calls for halving the proportion of the global population without sustainable access to safe drinking water between 1990 and 2015. Although this target was met in 2010, according to latest figures, 768 million people world-wide still rely on unimproved drinking water sources. Access to clean drinking water is integral to good health and a key strategy in reducing diarrhoeal illness: Currently, 1.3 million children aged less than five years die of diarrhoeal illnesses every year with a sixth of such deaths occurring in one country—India. Although India has recently made substantial progress in improving water supplies throughout the country, currently almost 90% of the rural population does not have a water connection to their house and drinking water supplies throughout the country are extensively contaminated with human waste. A strategy internationally referred to as Household Water Treatment and Safe Storage (HWTS), which involves people boiling, chlorinating, and filtering water at home, has been recommended by the World Health Organization and UNICEF to improve water quality at the point of delivery.

**Why Was This Study Done?:**

The WHO and UNICEF strategy to promote HWTS is based on previous studies from low-income settings that found that such interventions could reduce diarrhoeal illnesses by between 30%–40%. However, these studies had several limitations including reporting bias, short follow up periods, and small sample sizes; and importantly, in blinded studies (in which both the study participants and researchers are unaware of which participants are receiving the intervention or the control) have found no evidence that HWTS is protective against diarrhoeal illnesses. So the researchers conducted a blinded study (a double-blind, randomized placebo-controlled trial) in Orissa, a state in southeast India, to address those shortcomings and evaluate the effect of household water treatment in preventing diarrhoeal illnesses in children under five years of age.

**What Did the Researchers Do and Find?:**

The researchers conducted their study in 11 informal settlements (where the inhabitants do not benefit from public water or sewers) in the state's capital city and also in 20 rural villages. 2,163 households were randomized to receive the intervention—the promotion and free distribution of sodium dichloroisocyanurate (chlorine) disinfection tablets with instruction on how to use them—or placebo tablets that were similar in appearance and had the same effervescent base as the chlorine tablets. Trained field workers visited households every month for 12 months (between December 2010 and December 2011) to record whether any child had experienced diarrhoea in the previous three days (as reported by the primary care giver). The researchers tested compliance with the intervention by asking participants if they had treated the water and also by testing for chlorine in the water.

Using these methods, the researchers found that over the 12-month follow-up period, the longitudinal prevalence of diarrhoea among children in the intervention group was 1.69% compared to 1.74% in the control group, a non-significant finding (a finding that could have happened by chance). There was also no difference in diarrhoea prevalence among other household members in the two groups and no difference in weight for age z scores (a measurement of growth) between children in the two groups. The researchers also found that although just over half (51%) of households in the intervention group reported treating their water, on testing, only 32% of water samples tested positive for chlorine. Finally, the researchers found that water quality (as measured by thermotolerant coliforms, TTCs) was better in the intervention group than the control group.

**What Do These Findings Mean?:**

These findings suggest that treating water with chlorine tablets has no effect in reducing the prevalence of diarrhoea in both children aged under five years and in other household members in Orissa, India. However, poor compliance was a major issue with only a third of households in the intervention group confirmed as treating their water with chlorine tablets. Furthermore, these findings are limited in that the prevalence of diarrhoea was lower than expected, which may have also reduced the power of detecting a potential effect of the intervention. Nevertheless, this study raises questions about the health impact of household water treatment and highlights the key challenge of poor compliance with public health interventions.

**Additional Information:**

Please access these websites via the online version of this summary at http://dx.doi.org/10.1371/journal.pmed.1001497.

- The website of the World Health Organization has a section dedicated to household water treatment and safe storage, including a network to promote the use of HWTS and a toolkit to measure HWTS
- The Water Institute hosts the communications portal for the International Network on Household Water Treatment and Safe Storage

## Introduction

Diarrhoea is responsible for an estimated 1.3 million deaths among children under five each year, mostly in developing countries [1]. With over 287,000 deaths attributable to diarrhoeal diseases per year, India ranks first among countries contributing to this worldwide disease burden [1].

India has made considerable progress in recent years in improving water supplies in both rural and urban settings [2]. Nevertheless, only 11% of the rural population is served by a household water connection. Surveys of microbial water quality throughout India have shown extensive faecal contamination of drinking water supplies. In Hyderabad, for example, 50% of water samples drawn from pre-monsoon, monsoon, and post-monsoon period were positive for faecal coliforms [3]. In Madhya Pradesh, 33% of boreholes were faecally contaminated [4]. Even water that is safe at the point of distribution is subject to frequent and substantial contamination during collection, transport, and storage [5].

Household water treatment and safe storage (HWTS), including boiling, chlorinating, and filtering water at home, can improve water quality at the point of delivery and prevent post-collection contamination. Systematic reviews of water quality interventions have shown HWTS to be effective in improving drinking water quality and in preventing diarrhoea [6]–[8]. Based on this evidence, the WHO and UNICEF recommend HWTS for populations relying on unsafe water supplies as part of a comprehensive strategy to prevent diarrhoeal disease, particularly among young children [9].

The evidence supporting a health impact from HWTS in low-income settings is from studies employing open trial designs. While open trials have found HWTS interventions to reduce diarrhoea within the range of 30%–40% [7],[8],[10], none of the blinded trials to date in low-income settings have found the intervention to be protective against diarrhoea [11]–[13]. However, previous studies presented certain limitations including good ambient drinking water quality, short follow-up periods, and small sample sizes. This disparity in results and the limited evidence from previous blinded trials has led researchers to call for more rigorous studies to quantify the contribution, if any, of HWTS in reducing diarrhoea [8],[14]–[16]. We undertook this study to evaluate the effect of household water treatment in preventing diarrhoea prevalence among children under five.

## Methods

### Ethics

The protocol was approved by the Ethics Committee of the London School of Hygiene and Tropical Medicine, and the Ethics Committee of the Indian Institute of Health Management Research. Written consent was obtained from all heads of households after being given full details about the study. Children reported to have diarrhoea at the time of visit were given oral rehydration sachets. When required, they were referred to the local community health worker or health centre. Following the conclusion of the study, results were shared with participating households. Each household was given a supply of oral rehydration solution and a 1-y supply of active tablets.

### Study Setting

The study was conducted in Orissa, India among 11 informal settlements in the capital city of Bhubaneswar and 20 rural villages in the district of Dhenkanal. Partly because they are considered squatters on public land, residents of the informal settlements are provided no water, sewer, or other public services. Drinking water is procured mainly from hand-dug wells, many of which are open and unprotected or from boreholes and tap stand. Dhenkanal district is located about 100 km northeast of Bhubaneswar and is inhabited mostly by agricultural labourers and workers at local steel plants. Households rely on poorly protected open hand-dug wells, or from yard or public taps connected to a distribution system drawing from wells. Open defecation is common among the study population.

### Eligibility and Enrolment

In November 2010, a community meeting was held in each site to explain the objectives of the study. Households were eligible to participate in the study if they had at least one child under 5 y of age and lived permanently in the selected area. Eligibility was verified with children's immunization records. Participating households were explicitly encouraged to continue their existing water treatment practices rather than rely on a tablet that may be a placebo. A baseline questionnaire was administered to each enrolled household to collect information on demographics, socio-economic characteristics, and water, hygiene and sanitation conditions and practices.

### Intervention

The intervention was implemented by Population Services International (PSI), a leading promoter of chlorine-based HWTS products worldwide. The intervention consisted of the promotion and free distribution of sodium dichloroisocyanurate (NaDCC) disinfection tablets (Medentech, Ltd.). NaDCC tablets have long been used for the emergency treatment of water and more recently for the routine treatment of drinking water; they have been reported to offer some advantages over sodium hypochlorite in terms of safety, shelf life, up-front cost, and convenience [17]. This product was selected for the intervention because chlorine is widely used for water treatment and because a previous study reported that blinding of NaDCC tablets was feasible [11].

Prior to the commencement of the study, extensive piloting was conducted to determine the optimal dosing of the tablets based on chlorine demand of the water in the study area. While most study households were using a 13 l aluminium container to store their drinking water, it was necessary to use a 67 mg NaDCC tablet (normally designed to treat 20 l of water) in order to achieve the WHO standard of a minimum of 0.2 ml/l of residual free chlorine (RFC) after 24 h [18]. In accordance with the manufacturer's instructions, households were advised to add the tablet to their water storage container, stir or agitate it, and wait for 30 min prior to consumption of the treated water.

A team of 20 trained inter personal communicators (IPCs) employed by PSI visited each household fortnightly and gave them free of charge a box containing 30 tablets along with instructions on use. During each visit, IPCs also provided information on the adverse health effects associated with consuming contaminated drinking water and the importance of treating drinking water. Games and interactive pictures were used to engage household member in the discussions. Community-level activities such as street plays, game shows, wall paintings, and distribution of fliers, posters, and calendars were also conducted throughout the study.

### Randomisation and Blinding

Following baseline, households were randomly assigned to one of the two study arms. Randomisation was stratified by community to ensure an equal number of intervention and control households in each of the 31 rural and urban communities. The randomisation list was generated using Stata 10 and was conducted by a member of staff who was neither involved in the delivery of the intervention nor in the data collection. The allocation sequence was concealed from the implementing team and the evaluation team. The placebo tablet was similar in appearance to the active tablet. It had the same effervescent base but did not contain the NaDCC disinfecting agent. The active and placebo tablets were packaged in identical boxes of three strips containing ten tablets each. Each box was pre-labelled with the allocated household identification number prior to distribution. The labeling of the boxes was conducted by members of staff who were neither involved in the implementation nor data collection or analysis. Boxes of active and placebo tablets were labelled with a distinct batch code generated by the manufacturer and only known to the member of staff responsible for supervising the labeling of the boxes with the household identification numbers.

### Outcome Assessment

The primary outcome was longitudinal prevalence (LP) of diarrhoea among children under 5 y of age. Longitudinal prevalence was recorded as the number of days with diarrhoea over the total number of days under observation. This outcome was chosen because it is reported to be more closely associated with mortality [19]. Diarrhoea among participants of all ages was recorded as a secondary outcome. Trained fieldworkers visited households every month for 12 mo (between late December 2010 and December 2011). At each visit, daily point prevalence over the previous 3 d (today, yesterday, and the day before yesterday) was recorded for each child <5 y based on reports from the primary care giver [20]. Follow-up resulted in 12 visits and 36 possible days of observation. Diarrhoea was defined using the WHO definition of three or more loose stools passed in one day [21].

Weight-for-age z-score (WAZ), school absenteeism, and health care expenditure for diarrhoea were included as secondary outcomes. WAZ was measured as a potential proxy marker for recent diarrhoea [22]. WAZ was measured among all children under five at baseline and each of the monthly visits. Weight was measured using a portable digital scale SECA 385 with an increment of 20 g for weight below 20 kg and 50 g for weight between 20–50 kg. Field workers were trained and followed standardised procedures [23]. To monitor accuracy of the measurements, 10% of weight measurements were repeated over the course of the study [24]. WAZ were calculated using WHO growth reference data [25].

School absenteeism was assessed among primary school-aged children. Information on school asbenteeism was collected through caregivers' reports, roll calls, and review of attendance records at the schools. At each monthly visit, the mother or primary caregiver was asked if her child had been absent on any given day of school during the previous 5 d of school. Roll calls in the classroom were conducted in a total of nine visits over the course of the study. Schools to be visited for roll calls had to include at least ten children to be visited in order to facilitate logistics. School registers were reviewed for the entire follow-up period.

### Adherence

Adherence to the intervention was assessed by asking householders if they had treated their children's drinking water and by testing it for RFC. If the caregiver replied that the child's drinking water was treated, she was asked which method was used. This open-ended question was designed to monitor not only reported use of tablets, but also of other water treatment methods. RFC concentrations were measured by colorimetric method using DPD1 reagent (Palintest Limited, Tyne & Wear) and a colour comparator. The scale of the comparator allowed for readings by 0.1 mg/l from 0.1 to 1.0 plus eight readings between 0.5 and 6.0 mg/l. Chlorine testing was done immediately after sample collection but off site and by a separate fieldworker to preserve blinding. Householders who reported their water to be treated with a tablet at the time of visit were defined as reported users while those with detectible RFC in the sample were defined as confirmed users.

### Water Quality

Each month, 20% of households were randomly selected for testing of children's drinking water for the presence of thermotolerant coliforms (TTC), an indicator of faecal contamination [18]. Samples were collected directly from the vessel containing water being consumed by the child <5 y using sterile 125 ml Whirl-Pak bags (Nasco) containing a tablet of sodium thiosulfate to neutralise any halogen disinfectant and were placed on ice for transport to the laboratory. Samples were processed within 4 h using the membrane filtration technique on a 0.45-micron membrane (Millipore Corporation), cultured on membrane lauryl sulphate medium (Oxoid Limited), and incubated at 44°C [26]. For quality control, 10% of samples were processed in duplicate and a negative control was processed with each batch.

### Blinding Assessment

On the last follow-up visit the mother or primary caregiver of the child was asked to guess whether they had been receiving the active tablet or placebo. Success of blinding was measured using both the James' and Bang's blinding indices [27],[28].

### Statistical Analysis

The sample size was calculated assuming a 15% reduction in the longitudinal prevalence of diarrhoea among children of the intervention arm [29],[30]. We assumed 80% power, significance level 2-sided α = 0.05, a baseline diarrhoea prevalence of 4%, a standard deviation of 4.4%, a design effect of 1.1 for clustering of diarrhoea cases at the household level, and 15% loss-to follow-up. The sample size was increased to account for intermittent sampling and a 3-d recall period [31]. Calculations yielded a sample size of 1,500 children <5 y per arm. Assuming 1.5 children <5 y per household, the number of household to be recruited was 1,000 per arm.

The primary measure of efficacy of the intervention was estimated using a intention-to-treat analysis. By intention-to-treat, we mean that participants were analysed in the treatment groups to which they were randomised, irrespective of the intervention they actually received [32]. However, the analysis does not include missing outcome data resulting from lost-to-follow-up or participants who remained in the study until the end of the follow-up period, but who were absent on some of the visits. All statistical analyses were conducted in Stata 10. We estimated the prevalence ratio of diarrhoea using binomial regression with a log link function and robust standard errors. We used generalised estimating equations (GEE) with an exchangeable correlation matrix to account for clustering at the household level [33]. GEE has been suggested as an appropriate method to account for clustering since it relies on fewer assumptions than random effect models for binary outcomes [34].The effect of the intervention on WAZ was assessed using random effect linear regression adjusting for baseline WAZ measurement. In a deviation from the study protocol, we explored the within-child effect of diarrhoea on WAZ using fixed effect linear regression with number of days with diarrhoea in the previous 3-d window as a categorical variable [35]. The fixed effect model was deemed more appropriate to control for any confounding due to differences between individual children.

Statistical analyses of microbiological data were conducted after log10 transformation of TTC counts to account for the skewed distribution. Geometric mean TTC counts were reported in each treatment arm. Means of the log-transformed values were compared between groups using non-parametric tests. Subgroup analyses were conducted to assess the effect of the intervention among the treated. We compared prevalence of diarrhoea and faecal contamination levels among self-reported users of the control and intervention groups.

## Results

### Participants

**Figure 1:**
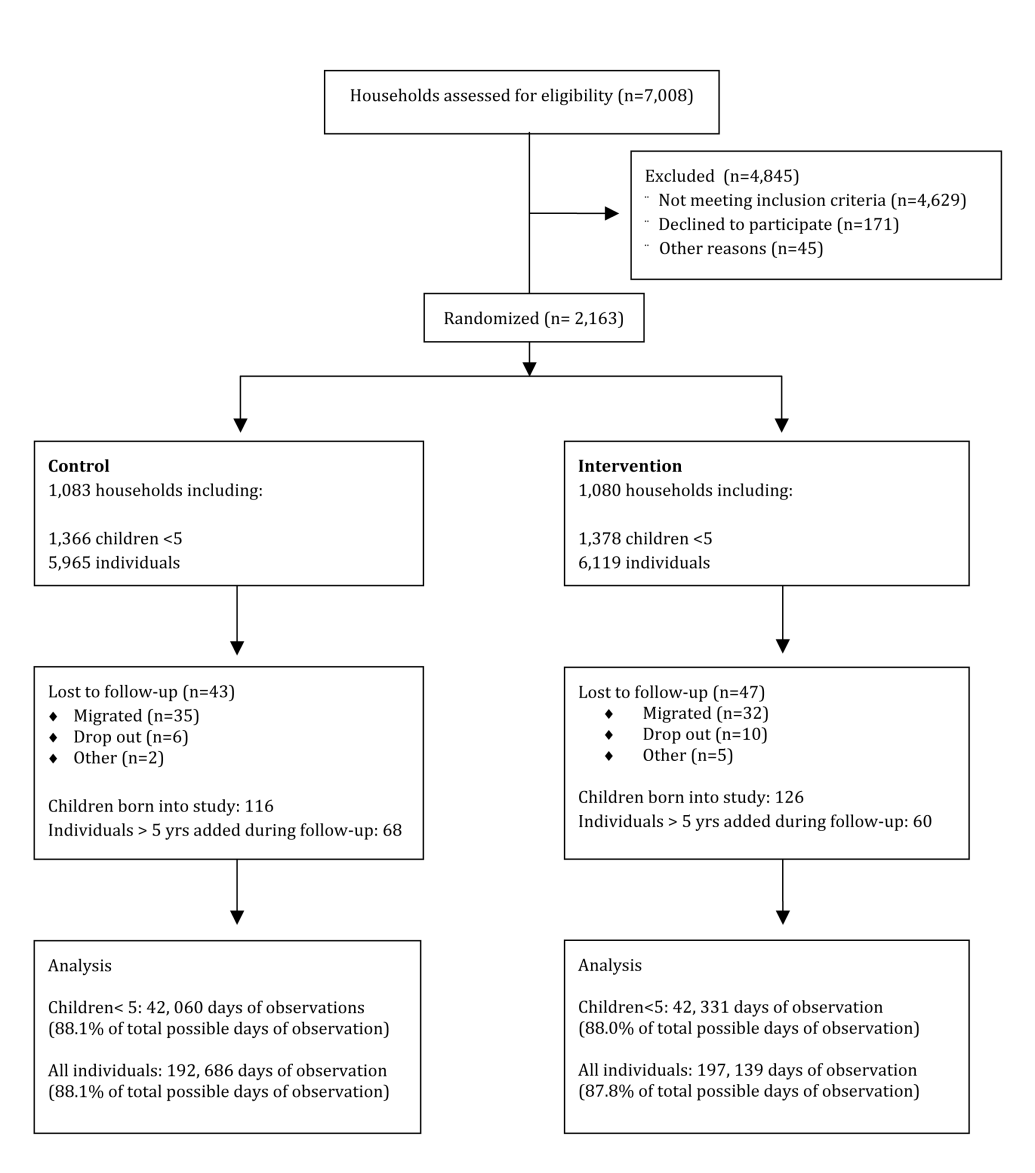
Trial profile.

Overall, 2,163 households and 12,084 individuals were enrolled in the study. This number includes 2,744 children under 5 y. An additional 242 children were born during the 12-mo follow-up period, and 128 household members who were missed during the census at baseline were included from the first round of follow-up. A total of 46 individuals died over the course of the study, three were children <5 y (two due to accidents, one cause of death not specified) (Figure 1). Over the follow-up period, data were obtained on 84,391 d of observation for children <5 y and 389,825 d of observation for participants of all ages, representing 88% of total possible days of observation. Observations were missing due to household members moving out of the study area or being absent at the time of visit. In per-visit analysis of missing data, including participants who remained in the study but missed visits, the proportion of missed visits was neither associated with treatment arm (*p* = 0.94) nor water treatment practices at baseline (*p* = 0.69) and age (*p* = 0.83). However, it was higher among male than female (12.5% versus 11.2%, *p*<0.01) and among those who had diarrhoea at baseline (17.2% versus 11.5%, *p*<0.001).

### Baseline Characteristics

Baseline characteristics were well balanced between groups (Table 1). Overall, 44% of households reported treating their drinking water. Of those, boiling was the most common method (68%) followed by straining water through a cloth (11%). Water was typically stored in wide neck aluminum containers (81%). Half of household reported dipping a cup or using a long ladle to serve water from the container.

**Table 1:** Baseline characteristics of study households (*n* = 2,163).

Characteristics	Control		Intervention	
	*n*	Percent	*n*	Percent
**Demographic and socio-economic**				
Number of households	1,083	50.1	1,080	49.9
Urban	340	31.4	338	31.3
Rural	743	68.6	742	68.7
**Mean (SD) number of persons per household**	5.5 (2.2)		5.7 (2.3)	
**Education head of household**				
Illiterate	206	19.0	188	17.4
Literate no formal schooling	69	6.4	90	8.3
Some primary	139	12.9	160	14.8
Completed primary	160	14.8	146	13.5
Some secondary	387	35.8	400	37.0
Completed +2 y	65	6.0	48	4.4
Completed +3 y (university)	56	5.2	48	4.4
**Mean (SD) number of rooms for sleeping**	1.7 (1.0)		1.7 (1.0)	
**Own**				
Electricity	824	76.1	821	76.1
TV	554	51.2	549	50.9
Refrigerator	125	11.5	124	11.5
Bicycle	762	70.4	748	69.3
Motorbike	250	23.1	247	22.9
Land	540	49.8	552	51.1
Livestock	452	41.8	464	43.0
**Type of construction**				
Pucca	429	39.7	398	36.9
Semi-pucca	285	26.3	286	26.5
Kuchha	268	34.0	396	36.7
**Drinking water source - rainy season**				
Unprotected dug well	667	61.7	672	62.2
Tubewell	187	17.3	175	16.2
Tap	147	13.6	138	12.8
Surface water	53	4.9	58	5.4
**Drink from same water source during dry season**	946	87.3	951	88.1
**Type of container**				
Wide neck	881	81.5	884	82.1
Narrow neck	36	3.3	28	2.6
Both types	164	15.2	165	15.3
**Serving child's water**				
Dip cup	542	50.4	508	47.3
Pour	488	45.4	523	48.7
Use tap	34	3.2	28	2.6
**Treat water**	482	44.6	470	43.6
**Treatment method**				
Boil	319	66.2	331	70.3
Strain	122	25.3	106	22.5
Chlorine	11	2.3	9	1.9
Other	30	5.0	24	3.8
**Toilet facilities**	422	39.0	410	38.0
**Use soap to wash hands**	372	34.4	330	30.6
**Hand washing station**	659	60.9	658	60.9
Water present	596	90.4	595	90.6
Soap present	317	48.1	314	47.8
Bucket present	522	79.5	533	81.4
**Faeces present in courtyard**	475	43.9	453	41.9

SD, standard deviation.

### Diarrhoea and Weight-for-Age

**Figure 2:**
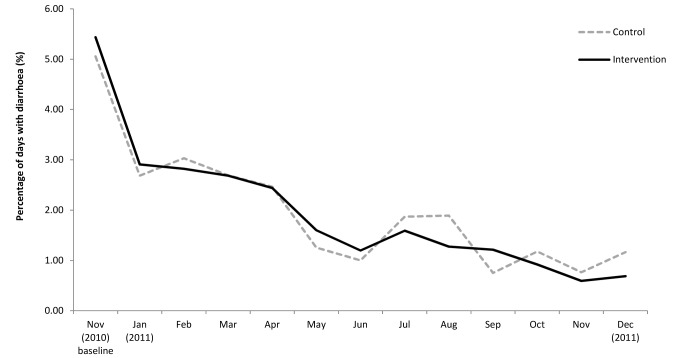
Prevalence of diarrhoea among children <5 y over time.

Over the 12-mo follow-up period, the longitudinal prevalence of diarrhoea among children of the intervention group was 1.69% compared to 1.74% among the control group. After adjusting for clustering within the household, the longitudinal prevalence ratio was 0.95 (95% CI 0.79–1.13) (Table 2). Among participants of all ages, the longitudinal prevalence ratio was 0.99 (95% CI 0.84–1.15). Prevalence of diarrhoea declined over time in both treatment arms ranging from 5.20% at baseline to 0.92% on the last round of follow-up among children under five (Figure 2) and from 1.90% to 0.31% among participants of all ages. When time was included in the model, we found no evidence of an interaction between time and treatment arm (*p* = 0.67). The effect of the intervention did not change over the course of the follow-up period. We found a design effect of 1.1 for clustering of diarrhoea among children <5 y within the same household.

**Table 2:** Longitudinal prevalence of diarrhoea among children under five and individuals of all ages by treatment arm and stratified by reported use.

Analysis	Control			Intervention			LPR Adjusted^a^
	Days with Diarrhoea	Days of Observation	LP (%)	Days with Diarrhoea	Days of Observation	LP (%)	
**Intention-to-treat analysis**							
<5	733	42,060	1.74	715	42,331	1.69	0.95 (0.79–1.13)
All ages	1,172	192,686	0.61	1,163	197,139	0.59	0.99 (0.84–1.15)
**Subgroup analysis stratified by reported use**							
<5							
User	360	25,157	1.43	310	21,122	1.47	1.02 (0.80–1.30)
Non-user	352	16,367	2.15	391	20,568	1.90	0.88 (0.70–1.12)
All ages							
User	574	114,361	0.50	507	95,256	0.53	1.08 (0.88–1.32)
Non-user	551	72,028	0.76	630	95,249	0.66	0.90 (0.73–1.10)

a LPR Adjusted for clustering within household.

Each additional day of diarrhoea resulted in a decrease of 0.0249 in WAZ (95% CI −0.0365 to −0.0133). However, the intervention had no effect on WAZ (mean WAZ of −1.589 among control children versus −1.586 among intervention). The regression coefficient after adjusting for baseline WAZ was 0.0003 (−0.0347; 0.0354).

### Adherence

**Figure 3:**
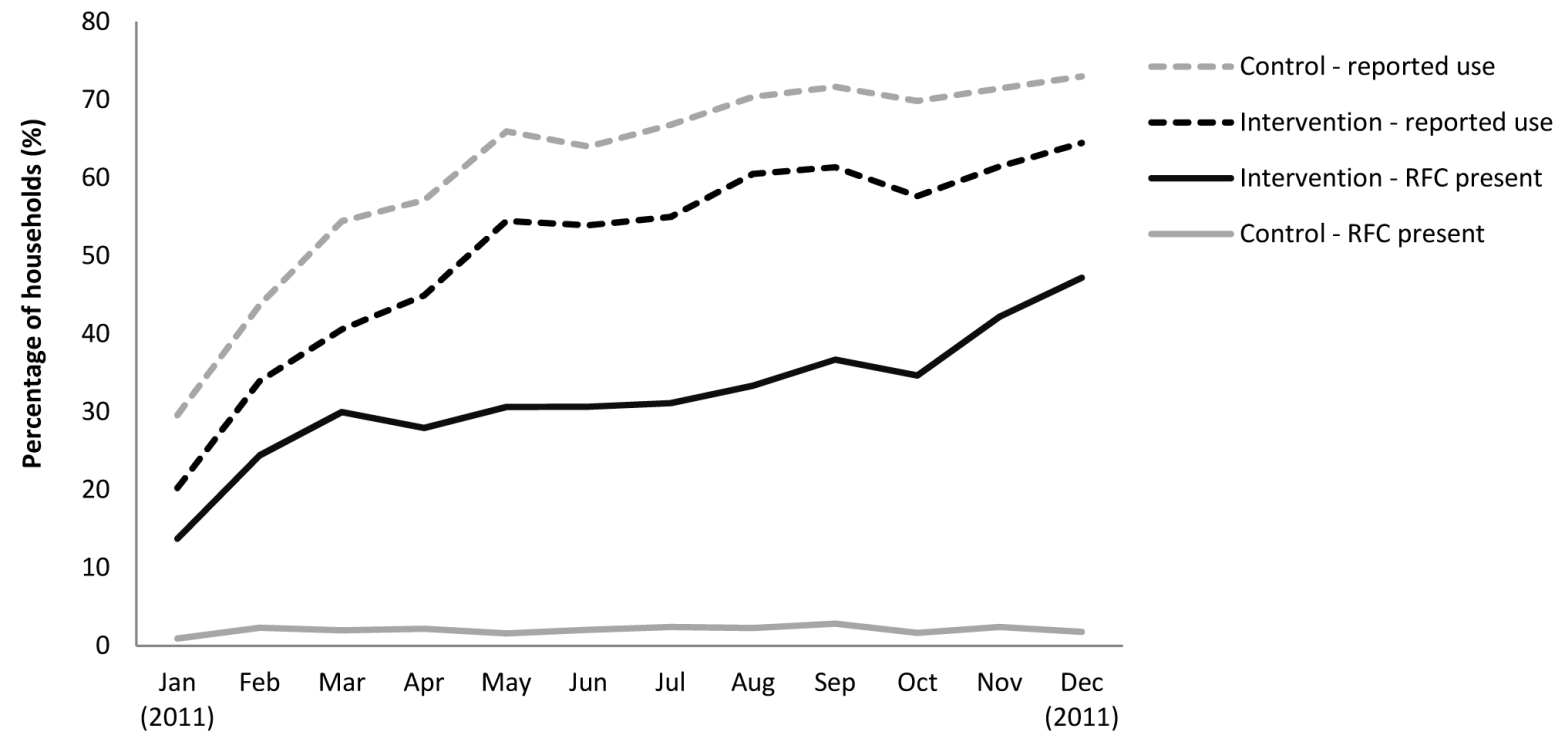
Compliance assessed by presence of residual chlorine in child's drinking water and self-reported use among intervention and control households over time.

Children's drinking water was tested for RFC on 88% of 25,956 total possible household visits. While 51% of intervention households reported their child's drinking water to be treated with the tablets at the time of visit, only 32% of the water samples tested positive for RFC. Reported use was higher among households who received the placebo (62%) compared to the active tablet (51%). Only 2% of control group samples had RFC (Table 3). In both groups, reported use of tablets increased over the 12-mo study period (Figure 3). Presence of RFC among intervention households also increased over time, ranging from 14% one month after the start of promotional efforts to 47% on the last month of follow-up. While the use of tablets increased over time, reported use of other water treatment methods declined.

**Table 3:** Adherence measured by presence of residual free chlorine (*n* = 22,804) and self-report (*n* = 22,976).

Adherence	Control			Intervention		
	*n*	Total	Percent	*n*	Total	Percent
RFC	223	11,407	2	3,630	11,397	32
Self-report	7,071	11,485	62	5,829	11,491	51

Overall, 20% of the 1,080 intervention households never had residual chlorine in their child's water during follow-up visits and 76% had chlorine on less than half of the total visits. Of the 3,863 samples that tested positive for chlorine, 70% (2,727) had RFC concentrations that fell within the CDC Safe Water Program recommended thresholds for acceptable taste and odour (≤2.0 mg/l). However, 30% of the samples had concentrations above that level, including 11% (444) equal to or exceeding the WHO recommended maximum concentration of 5 mg/l [18].

### Water Quality

**Figure 4:**
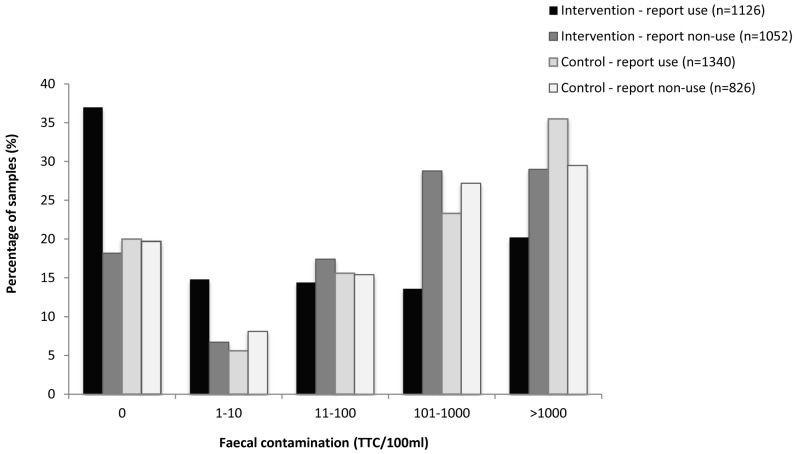
Faecal contamination levels in child's water samples by self-reported use (n = 4,344).

A total of 4,546 samples of child's water were tested for thermotolerant coliforms (TTC/100 ml), representing 18% of the total number of household visits. Faecal contamination of drinking water was lower among intervention households than controls (geometric mean TTC count of 50 [95% CI 44–57] per 100 ml compared to 122 [95% CI 107–139] per 100 ml among controls [*p*<0.001]). Reported users in the intervention arm had lower TTC counts (geometric mean was 24, 95% CI 20–29) compared to those in the control arm (geometric mean 138, 95% CI 116–162 [*p*<0.001]). Overall, 37% (417/1,126) of samples of reported users in the intervention arm had no detectible thermotolerant coliforms at the time of visit against 20% (268/1,340) in the control arm (Figure 4). Over the study period, a total of 621 water samples were processed in duplicate for quality controls. The intraclass correlation coefficient calculated on the log-transformed values of TTC counts indicated good reliability between duplicate measurement, with an ICC of 0.82, 95% CI (0.69–0.96).

### Diarrhoea and Adherence

The prevalence of diarrhoea was significantly lower among children from families confirmed to be using the tablet than those who did not, irrespective of treatment arm (1.23% versus 1.78%, respectively) with a prevalence ratio of 0.72 (95% CI 0.57–0.91). However, this analysis may exaggerate the effect since users and non-users may differ with respect to certain characteristics affecting diarrhoea. This is confirmed by the observation that even in the control arm, reported users had less diarrhoea than non-users.

A comparison between users across treatment is likely to be less biased. We estimated the average effect of treatment among reported users since laboratory confirmation of placebo tablet use was not possible to obtain from the control group. In the intervention arm, reported users had residual chlorine in their water in 60% (3,440/5,784) of samples taken (versus only 3% [175/7,036] among reported non-users). Longitudinal prevalence of diarrhoea among children of households reporting use of the tablet in the intervention arm was the same compared to those of the control (1.47% versus 1.43%, respectively). The risk ratio was 1.02 (95% CI 0.80–1.30). A similar pattern was observed among members of all ages (Table 2).

### Diarrhoea and Water Quality

We explored the relationship between risk of diarrhoea and levels of faecal contamination in the child's drinking water. Children under five drinking water with TTC levels >1,000 per 100 ml did not have an increased risk of diarrhoea compared to TTC levels <1,000 TTC/100 ml (LPR 1.12, 95% CI 0.84–1.49). A similar result was observed among participants of all ages.

### School Absenteeism

At baseline, 1,059 children (5–10 y) were reported to attend primary school (grade 1 to 5) at 104 different schools. Overall, 34 schools had 10 or more pupils. Of those, it was possible to visit schools and conduct roll calls from 25 schools. School absenteeism information through roll calls and school registers was obtained for 611 students. Overall, 30% of primary care givers reported that their child missed at least 1 d of school during the previous 5 d of school (Table 4). On roll-call days, 21% of children were absent at the time of the school visit; this estimate was higher than the 14% school absenteeism figure obtained from the school registers. Prevalence of school absenteeism assessed by caregiver's report and review of school records was similar between treatment groups.

**Table 4:** School absenteeism among school-aged children assessed via mother's report, classroom roll calls and school records.

Absenteeism	Control			Intervention			*p*-Value
	*n*	Total	Percent	*n*	Total	Percent	
Reported^a^	1,412	4,641	30	1,406	4,600	31	p = 0.36
Roll-calls^b^	437	2,253	19	474	2,047	23	p = 0.02
School records^c^	6,896	48,014	14	6,253	43,932	14	p = 0.78

a Reported: numbers and proportions of children who missed at least 1 d of school in the past 5 d of school (*n* = 1,059 children 5–10 y enrolled in primary school standard 1–5 at baseline followed up for 12 mo).

b Roll call: number of days absent over total number of days of observation (*n* = 611 children followed up for 9 mo).

c School records: number of days absent over total number of school days (*n* = 611 children followed up for 12 mo).

### Blinding

When asked to guess whether they had the active tablet or the placebo, 71% (669/936) of intervention households and 71% (684/961) of control households guessed they had the active tablet (Table 5). When the 25% who responded “don't know” were encouraged to guess, the proportions were 90% (852/936) and 91% (864/961), respectively. The blinding index calculated with James' method was 0.62 (0.61–0.63). The blinding index calculated from Bang's method was 0.69 (0.66–0.72) among participants who received Aquatabs and −0.68 (−0.70 to −0.65) among those who received the placebo.

**Table 5:** Blinding status of respondents by group assignment at the end of the study (*n* = 1,897).

Guess	Assignment					
	Placebo		Chlorine		Total	
	*n*	Percent	*n*	Percent	*n*	Percent
Chlorine	684	71.2	669	71.5	1353	71.3
Placebo	33	3.4	24	2.5	57	3.0
Don't know	244	25.4	243	26.0	487	25.7
Total	961	100	936	100	1897	100

At the end of the study, when asked about taste and smell of their drinking water after adding the tablet, 51% (483) of the 947 intervention households interviewed reported that the smell of the water was worse than untreated water compared to 23% (225/961) of control households. In addition, 22% (209) of intervention households complained about the taste of the water compared to 7% (72) among controls.

## Discussion

We conducted a large double-blind randomised controlled trial to assess the impact of household water treatment on diarrhoea among children <5 y in rural and urban India. Our findings provide no evidence that the intervention was effective in preventing diarrhoea, either among children <5 y or among all members of the study population. Neither was there evidence of an impact of the intervention on WAZ.

The study was designed to address some of the limitations of previous double blinded trials. First, the study was powered to measure impact among children under five, who are most vulnerable to diarrhoea. The sample population was ten times the size of previous blinded trials of household water treatment conducted in low-income settings [11]–[13]. Second, both urban and rural populations were included in order to increase generalisability of our findings. Third, the 1-y follow-up period was designed to account for seasonability and for reduced compliance over time as previously reported [7],[29]. Fourth, we measured WAZ, a potential proxy for self-reported diarrhoea. Lastly, adherence and water quality were also monitored extensively every month. Our findings are consistent with other blinded studies of water quality interventions that found no impact on diarrhoea [11]–[13]. However, there are alternative explanations for the observed lack of impact.

Adherence to the intervention was low. Despite free distribution of the tablets and an intensive promotion campaign, only a third of intervention households met the definition of confirmed users in any month during the follow-up period; three-quarters had chlorine on less than half of the total visits. Systematic reviews and modelling studies of water quality interventions have shown that the protective effect from HWTS interventions is reduced when adherence is low [7],[8],[36],[37]. However, other non-blinded studies of HWTS with comparable levels of adherence (around 30%) have nevertheless reported lower diarrhoea rates among intervention participants [30],[38]. As with many other open trials reporting on subjective outcomes such as diarrhoea, those estimates may have been overestimated due to reporting bias [14],[29].

Comparison was made between self-reported users of the intervention group with those of the control. While householders exaggerated reported use, confirmed users nevertheless accounted for most (60%) of reported users. Although reported users of the intervention group had significantly less contaminated water than those of the placebo group, they did not have a lower prevalence of diarrhoea. This result speaks against low compliance being the only explanation for the lack of impact.

The low level of uptake was unanticipated. A 5-wk pilot of the same intervention among a comparable study population conducted immediately before the trial resulted in 68% compliance and greater than 2 log reduction in thermotolerant coliform counts. A number of chlorine-based interventions have achieved compliance in excess of 80% and a previous trial of NaDCC tablets in Bangladesh reported nearly full compliance [7],[39]. Uptake did increase over time, and it is possible that the low uptake at the start was due in part to challenges in scaling up the promotional campaign. While the distribution of the tablets to all study households started the first month of follow-up, the community mobilisation activities were not fully rolled out until the second quarter of follow up.

These results highlight the challenges of acheiving high levels of uptake of the intervention despite an intensive campaign. Evaluations of other HWTS strategies in programmatic settings have also reported low levels of adoption [30],[40],[41], while research-driven studies have found higher levels of reported use [42]. Further research is needed to better understand how to achieve consistent and sustained adoption of these interventions on a programmatic basis and over the long term.

Another potential explanation for the lack of health effect was the comparatively modest improvement in water quality among intervention households. With a mean of 50 TTC/100 ml, even water sampled from intervention households would be classified as “moderate risk” using WHO nomenclature. Other studies of household water treatment have reported higher baseline levels of contamination and larger reduction in faecal contamination of drinking water from the intervention [16],[43],[44].

Although we cannot rule out the possibility that this modest improvement in water quality reduced the potential for health impact among our study population, subgroup analysis found no evidence of a dose-response relationship between water quality and diarrhoea. Other studies have also reported weak associations between levels of indicators of faecal contamination and risk of diarrhoea [45],[46].

Our study had certain limitations. The prevalence of diarrhoea among children <5 y was lower than expected. As found in many other studies of water, sanitation, and hygiene (WASH) interventions, diarrhoea dropped significantly over time in both control and intervention groups. The lower prevalence may have also reduced power for detecting a potential effect. The amount of missing data was not unusual compared with similar trials. The propotion of missing visits was similar between treatment arms, but certain groups (female and those reporting no diarrhoea at baseline) were over-represented. Adjusting for covariates predicting missingness in the model did not change the effect estimate. Given the modest percentage of missing data in the study, any resulting bias is likely to be small.

The finding that children who experienced more diarrhoea in the previous 3 d had lower WAZ scores does provide support for this measure as a potential proxy marker for diarrhoea. More objective indicators are needed, especially for environmental health interventions that are difficult or impossible to blind.

Improvement in water quality alone may not be sufficient to prevent diarrhoea in settings with multiple sources of exposure to faecal pathogens. In this population, sanitation coverage and practices of handwashing with soap were low, indicating that other transmission routes may have played a more important role. Systematic reviews have reported subtantial reductions in reported diarrhoea from HWTS interventions alone, often with no additive effect from multiple intervention strategies [6],[47]. However, mathematical models have suggested that the protective effect of water quality interventions against diarrheoa is largely influenced by the level of hygiene and sanitation in the community[48].

In conclusion, our study sought to measure the impact of household water treatment among children under five in low income settings in the absence of reporting bias. The study was designed to address some of the shortcomings of previous trials. Our findings are consistent with other blinded studies of water quality interventions that found no impact on diarrhoea [11]–[13]. Both intention-to-treat analysis and analysis among reported users found no evidence of an impact on diarrhoea among children <5 y or all ages. Although we cannot rule out the possibility that this was due to low compliance and only a moderate impact of the intervention on water quality, our results raise additional questions about the protective effect of household water treatment under these conditions and underscore the need for promoters of household water treatment to demonstrate health impact in the absence of bias.

## Supporting Information

Text S1 **Study protocol.**
(PDF)

Text S2 **CONSORT checklist.**
(DOC)

## Abbreviations

HWTS: household water treatment and safe storage
NaDCC: sodium dichloroisocyanurate
RFC: residual free chlorine
TTC: thermotolerant coliform
WAZ: weight-for-age z score
