# Supplementary material for: Effect of Household-Based Drinking Water Chlorination on Diarrhoea among Children under Five in Orissa, India: A Double-Blind Randomised Placebo-Controlled Trial
Source: PLoS Med. 2013 Aug 20;10(8):e1001497. doi: 10.1371/journal.pmed.1001497 (PMC3747993; doi:10.1371/journal.pmed.1001497)
Supplement: Text S1 — Study protocol. (PDF) [file pmed.1001497.s001.pdf]

DOUBLE-BLINDED, RANDOMIZED, PLACEBO-CONTROLLED TRIAL TO ASSESS THE EFFICACY OF AQUATABS™ NADCC TABLETS TO PREVENT DIARRHOEAL DISEASE

STUDY PROTOCOL

---

1. BACKGROUND

1.1. Rationale for household water treatment

Diarrhoeal diseases kill an estimated 1.8 million people each year (WHO 2005). Children under 5 years of age are especially vulnerable, representing more than 90% of this disease burden. Among children under 5 in developing countries, diarrhoeal diseases account for 21% of all deaths (Parashar 2003). Although diarrhoea accounts for more than 4.2% of the worldwide disease burden (WHO 2004), it is largely preventable. Ingestion of unsafe water, along with inadequate availability of water for hygiene and a lack of sanitation, account for 88% of deaths from diarrhoea (WHO 2002). Expanding access to safe drinking water is among the chief priorities expressed in the UN's Millennium Development Goals (MDGs) (WHO/UNICEF 2008).

An estimated 884 billion people lack access to safe water (WHO/UNICEF 2008). The problem is particularly acute among rural and urban slum populations, many of which suffer from increased exposure due to poor sanitation. Even systems that qualify as "improved" under the WHO/UNICEF assessment, such as protected wells and gravity systems, often deliver highly contaminated water due to improper maintenance or operation (RDWQA 2006). Storing water in the home, a common practice due to distant or unreliable water supplies, is also a source of diarrheagenic pathogens (Wright 2004). As a result, the WHO has encouraged the development and use of improved point-of-use water treatment at the household level (WHO 2007). These interventions were to be an effective and cost-effective means of preventing diarrhoeal and other waterborne diseases (Fewtrell 2005, Clasen 2007).

In order to ensure the responsible deployment of such interventions, the WHO recommends that any products designed to treat water at the household level undergo independent testing to assess their microbiological effectiveness and health impact. The double-blinded, randomised, placebo-controlled trial is the gold standard of epidemiological evidence.

We propose to conduct such a trial to assess the impact of in-home chlorination on diarrhoeal disease among children under five in India. With nearly 400,000 deaths of children each year from diarrhoeal diseases, India alone accounts more than 20% of the worldwide mortality associated with diarrhoea (Unicef 2009). Despite considerable progress toward the MDG water target, large portions of the population, particularly in rural settings, still lack access to improved water supplies (WHO/UNICEF 2008). This population could therefore benefit from safe and effective household water treatment. Much of that population lives on less than $1 or $2 per day, rendering higher-cost solutions such as filters prohibitive; a simple, low-cost chlorine solution may be most appropriate in India.

The intervention consists of dichloroisocyanurate (NaDCC) (brand name "Aquatabs"). The tablets present certain cost, logistical and other advantages over other chlorine sources suitable for household use. While long used by UNICEF and others in emergency response, NaDCC has now been approved by the WHO and USEPA for routine treatment of drinking water.

1.2. Previous Field Trials

In 2006, Clasen and colleagues conducted a four-month double-blinded, randomised, placebo-controlled trial among 100 householders within an informal urban settlement in Bangladesh to assess the microbiological effectiveness of NaDCC tablets (Clasen 2007). Monthly samples of stored drinking water from intervention households were significantly lower in themotolerant coliforms (TTC) than those of control households (geometric mean 2.8 (95%CI: 2.2, 3.6) versus 604.1 (95%CI: 463.2, 787.9), P<0.0001). While none of the 191 samples from the control group met WHO guidelines for zero TTC/100ml, 61.7% samples from the intervention households met such benchmark. All 188 samples from intervention households were positive for residual free chlorine (compared to none of the control household samples), suggesting that householders consistently used the tablets.

The US Centers for Disease Control and Prevention (CDC) subsequently conducted a double-blinded RCT to determine the health impact of daily use of NaDCC tablets for household drinking water treatment in peri-urban Ghana (Jain 2010). They randomized 240 households (3,240 individuals) to receive either NaDCC or placebo tablets. All households received a 20-liter safe water storage vessel. Over 12 weeks, 446 diarrhea episodes (2.2%) occurred in intervention and 404 (2.0%) in control households (p=0.38). Residual free chlorine levels indicated appropriate tablet use. Escherichia coli was found in stored water at baseline in 96% of intervention and 88% of control households and at final evaluation in 8% of intervention and 54% of control households (p=0.002). NaDCC use did not prevent diarrhea but improved water quality. However, diarrhea rates were low and water quality improved in both groups.

The investigators concluded that a follow-up health impact study is warranted. Previous double-blinded trials of household-based water treatment interventions conducted in low-income settings also reported no protective effect on diarrhoeal disease (Kirchoff 1985; Austin 1993). However, each of these studies were conducted under circumstances that raised questions about their results (Clasen 2006). This was also true of the CDC trial in Ghana. There the microbiological water quality was comparatively high, so that the difference in faecal contamination between intervention and control groups was relatively low. Moreover, as the investigators acknowledged, the safe water storage vessels may have been protective. Improved storage vessels have been shown to minimize the recontamination of drinking water in the home, and thus reduce exposure to diarrhoeagenic agents (Wright 2004). Improved vessels alone have been associated with a 31% reduction in diarrhoeal disease (Roberts 2001). Thus, the study was essentially a comparison between two interventions rather than an intervention and control. In this respect, it may not have had sufficient statistical power to detect a difference in diarrhoea incidence between the two groups.

2. Study objectives

The primary objective of the study is to evaluate the efficacy of NaDCC tablets in preventing diarrhoea among children under 5 years of age. The secondary objectives of the study are to measure (i) the impact of the intervention on diarrhoea among study participants of all ages (ii) the impact on weight gain among children <5 years (WAZ is proposed as a proxy for diarrhoea) (iii) the efficacy of the tablets to improve the microbiological quality of drinking water (iv) the extent to which the tablets are used correctly and consistently by the study population (v) the acceptability of intervention for potential long-term use, (vi) impact on school absenteeism (vii) the cost-effectiveness of the intervention.

3. Methods

The epidemiological design is a parallel double-blinded, randomised, placebo-controlled trial intended to assess the efficacy of NaDCC tablets in reducing diarrhoea among children <5 years over a twelve-month follow-up period.

3.1. Pilot phase

The pilot study will be carried out in one village that will not be part of the actual trial and will involve approximately 100 households with children <5. We will conduct formative research using methods such as focus group discussions and in depth interviews to gain an understanding of local perceptions and practices with regards to water, hygiene and sanitation, and investigate local terminologies for diarrhoea. Households will be explained the objectives of the pilot study. Those willing to participate will be asked to give consent in writing prior to enrollment. Participating households will be randomly allocated to one of two groups. Like in the actual trial, half will receive the active tablets and the other half will receive the placebo. Each household will be followed up for a month. During that period, data collection tools will be piloted. This study will also allow us to confirm the suitability of the area according to key study parameters, explore use and acceptability of the tablets among the target population (chlorine dosage, acceptability of taste etc.) and evaluate the effectiveness of blinding. At the end of the month, in-depth interviews will be conducted with a sample of households to assess whether blinding was successful and if not, identify the reasons why. This will be complemented by a cross-sectional survey asking each mother or primary care giver to guess which tablets they have received. At the end of the pilot phase, a decision to proceed with the larger trial will be made if results show high acceptability and high level of use and if blinding of the intervention is found to be successful.

3.2. Site selection

The study will be conducted in both rural and urban communities in the State of Orissa, India. Study sites will be selected based on the following parameters: (i) most households in the community relying on surface water or other unimproved water supplies with mean thermo tolerant coliforms counts of 500 per 100ml or above (ii) low percentage of the population regularly treating their water before drinking it in some microbiologically effective manner (e.g. boiling); (iii) the community has sufficient quantity and access to water supplies, so that major deficiency is microbiological water quality; (iv) the host organization has on-ground staff and other logistical support (v) the population and community leaders desire to participate in the Study (vi) The site is easily accessible even during the rainy season.

3.3. Enrolment and baseline

A community meeting will be held in each selected village to explain the objectives of the study. For each village, a census of all households will be obtained. Households are eligible to participate in the study if they have at least one child less than five years of age and live permanently in the study area. The age of the child at enrollment will be checked against vaccination records when available. Informed consent will be sought from the heads of the households prior to enrolment. A baseline survey will be undertaken to collect information on demographics, socio-economic characteristics, and water, hygiene and sanitation conditions and practices. The analysis of the baseline data will allow verifying that the two groups are balanced in term of factors that may influence the outcome of interest.

3.4. Randomisation and blinding

Following baseline, households will be randomly assigned to one of the two groups. Randomisation will be conducted by a member of staff that is neither involved in the delivery of the intervention, nor in the data collection or analysis of the data. Randomisation will be stratified by village and will be done using a computer generated random sequence. The field team involved in the evaluation of the intervention will be different from the team delivering it. The evaluation team will be divided into two groups. One team will be responsible for conducting health surveys and anthropometric measurements. Another team will be responsible for water testing and the evaluation of compliance. During the whole duration of the study, the allocation sequence will be kept hidden from the distributors, the evaluation team and the data analysis team.

3.5. The Intervention

Aquatabs are tablets formed from sodium dichloroisocyanurate (NaDCC) and an effervescent base. Widely used for decades in household and commercial laundry bleaches, scouring powders and industrial and recreational water disinfection, NaDCC has recently found applications ranging from sanitizing medical instruments to cleaning of baby bottles and contact lenses. For more than 30 years, Aquatabs have been used for the emergency treatment of water. Its widespread use in non-emergency household water treatment applications began after acceptance of the chlorinated isocyanurates by the United States Environmental Protection Agency (USEPA), WHO and the Food and Agriculture Organization of the United Nations for the routine treatment of drinking water. NaDCC may also offer certain other advantages over sodium hypochlorite in terms of safety, shelf life, up-front cost and convenience, although these have not yet been shown to impact coverage or uptake (Clasen & Edmondson 2006).

Chlorine tablets such as Aquatabs may present a unique opportunity for blinding a household water treatment intervention in low-income settings. Tablets can be manufactured with the effervescent base and no NaDCC and packaged in a manner identical to Aquatabs. Other blinded trials conducted in low-income settings used liquid sodium hypochlorite that may have been noticeable to study participants due to the strong odour of the agent (Kirchoff 1985; Austin 1993). However, prior field trials of Aquatabs showed no evidence that intervention and control populations could detect whether they were using the tablets containing NaDCC (Jain 2010).

Each participating household will be provided with sufficient supplies of Aquatabs or placebos free of charge. Supplies of tablets at the household will be counted by the distributors every two weeks during their visit and topped off to cover the following two weeks. Households will also receive instructions on the use of the same in accordance with the manufacturers instructions and PSI's experience. Consistent with its normal practices, representatives of PSI will make regular visits to participating communities to ensure that they have adequate supplies. The study participants will be advised prior to the commencement of the study that half of the study households will receive a placebo. For this reason, they will be encouraged to continue

to follow their existing water treatment and handling practices and not to take any additional risk with respect to their drinking water management that might increase their exposure.

3.6. Outcome assessment

3.6.1. Diarrhoea morbidity

The primary outcome is longitudinal prevalence of diarrhoea, which is the number of days ill over the total number of days under observation. Longitudinal prevalence is chosen because it is reported to be closely associated with mortality (Morris 1996). The follow-up will be conducted monthly over 12 months. At each visit, the mother or primary care giver will be asked questions about diarrhoea today, yesterday and the day before yesterday for the child and for each family member who is absent at the time of visit. Diarrhoea will be defined using the WHO definition of three or more loose stools passed within a 24-hour period. The pilot will help identifying the local terms that most closely match that definition. Children found to suffer from diarrhoea will be given oral rehydration sachets and instructions on how to use them. If required, they will be referred to the closest community health centre to receive medical care free of charge.

3.6.2. Weight-for-age Z score (WAZ)

During health surveys, weight will be recorded for each child less than five years of age. Recent analyses suggest that there is a very strong association between weight-for-age Z score (WAZ) and diarrhoea over the last two weeks, which indicates that WAZ may be a good marker for recent diarrhoea (Schmidt 2009). Therefore, WAZ measured repeatedly in each child will be used as a proxy indicator of diarrhoea. WAZ will be measured according to a standard procedure and scales to minimise measurement error (Ulijaswek 1999, Cogill 2003). To check the accuracy of the measurements, a different member of the study team will repeat 10% of measurements on the same day.

3.6.3. Water quality

Thermotolerant coliforms will be used as an indicator of faecal contamination in drinking water samples (WHO 1993). Each month, a random sample of 20% of households will be selected. Field staff will conduct unannounced visits and take a sample from the storage vessel designated as containing the child's drinking water. All samples will be collected in sterile 100ml Whirl-Pak bags with a tablet of Thio-Sulfate to inhibit any disinfectant. Samples will be transported to the lab in coolers and analysed within 4 hours. Microbiological assessment will be performed using the membrane filtration technique on membrane lauryl sulphate medium (Oxoid Limited, Basingstoke, Hampshire, UK) using a DelAgua field incubator (Robens Institute, University of Surrey, Guilford, Surrey, UK) in accordance with the kit's instructions.

3.6.4. Compliance

Compliance will be assessed in two ways: 1) by asking the respondent (primary care giver of the child) if the child's drinking water has been treated and if so, the method that was used 2) by measuring presence of residual free chlorine in household water storage container. Chlorine testing will be conducted in a similar way in control and intervention households. Measuring chlorine in the control arm will help assessing neutrality of the placebo tablets and monitor any potential contamination due to exchange of the tablets between households. Concentration of free chlorine in the water will be measured to monitor dosage levels among intervention households. Chlorine will be assessed using a colorimetric indicator and DPD1 reagent (Palintest Limited).

3.6.5. Cost savings and school absenteeism

We will use surveys of participating households to collect information on health care expenditures for diarrhoea, both to determine any cost savings that might incur to households by using the intervention (reduced expenditures on drugs and healthcare) and as a further objective outcome associated with the intervention. We will also determine the impact of the intervention on absenteeism from school through household survey each month, roll calls in study village schools, and registers.

3.7. Blinding assessment

At the end of the follow-up period, female head of household or primary care giver will be asked to identify which tablet they had received. Success of blinding will be measured using two different approaches: James' blinding index is a variant of the kappa statistics and has been previously used in blinded trials of household water treatment (James et al 1996; Colford 2002). Bang's blinding index consists of estimating, for each treatment group, the proportion of correct guesses beyond chance (Bang et al. 2004).

3.8. Sample size calculations

Our primary outcome is the mean longitudinal prevalence of diarrhea, expressed as the number of weeks with diarrhea divided by the number of weeks under observation. The number of children < 5 required in each group was derived using the standard formulae for comparison of two means:

$n = (z_{\alpha/2} + z_{\beta})^2 (sd_0^2 + sd_1^2) / (m_0 - m_1)^2$

where m_0 and m_1 are the means longitudinal prevalence of diarrhoea among the control and intervention group, respectively and sd their standard deviation.

Based on the results from recent studies on household water treatment (Hunter 2009, Mausezahl 2009), we powered the study to detect a 15% reduction in the mean longitudinal prevalence of diarrhea among the intervention arm. Our calculation assumes 80% power, significance level α=0.05, mean longitudinal prevalence of diarrhoea of 4% among children < 5 years at baseline, a coefficient of variation (sd/mean) = 1.1, design effect of 1.1 for clustering of diarrhoea cases at the household level, 15% lost to follow-up. The sample size will be increased to account for 3-day recall period and intermittent sampling (Schmidt 2010). Based on the above assumptions, we estimated that we need at least 1500 children < 5 per arm (3000 in total). Assuming 1.5 <5s per household, the number of households to be recruited is approximately 1000 per arm. The sample size will be adjusted according to the baseline diarrhoea estimates obtained during the six-week pilot study.

3.9. Data Analysis

Data will double-entered in Epidata, will be checked for consistency and accuracy and will be analyzed in Stata 11.0 (Stata Corporation, College Station, Texas, US). The primary analysis is intention-to-treat; comparing the mean longitudinal prevalence of diarrhoea among children <5 in the intervention versus control groups. Diarrhoea among participants of all ages will also be reported. We will use generalized estimating equations (GEE) to calculate longitudinal prevalence ratio accounting for clustering at the household level. The effect of the intervention on WAZ will be assessed using random effect linear regression adjusting for baseline WAZ measurement. Any association between WAZ and number of days with diarrhoea in the previous 3 days will be explored using GEE with number of days included as a categorical variable. Statistical analyses of microbiological data are conducted after $\log_{10}$ transformation of TTC count values to account for the skewed distribution. Comparison between groups will be conducted using non-parametric tests if the distribution is not normal after log transformation. Subgroup analysis will be conducted to 1) measure the effect of compliance on diarrhea and 2) explore the relationship between faecal contamination levels and risk of diarrhoea.

3.10. Quality controls

Field staff will be extensively trained at the start of the trial. The training will cover basic epidemiological concepts and methods, ethics, interviewing techniques, weight measurement, water testing, data recording. Field supervisors will conduct unannounced random visits and re-interview 5% of respondents during the study period. 10% of weight measurements and 5% of water tests will also be done in duplicates. Regular meetings with the field team will be held to review progresses, discuss and resolve any issues encountered.

3.11. Ethics

Ethical approval will be sought from the ethics committee at the London school of Hygiene and Tropical Medicine and from the ethics committee at the Indian Institute for Health Management Research. The trials will be registered on http://clinicaltrials.gov/

4. Study Participants and Staffing

4.1. Study Participants

This study will be conducted by representatives of the London School of Hygiene & Tropical Medicine (LSHTM), a leader in research concerning water, sanitation and hygiene interventions in low-income settings. They will be working in collaboration with the Indian Institute of Health Management Research, a leading public health institute in India.

The intervention will be implemented by Population Services International (PSI), a US-based non-governmental organization with offices in New Delhi and extensive operations in India. PSI has broad experience with household water treatment interventions in 21 countries in Asia and Africa. It also has extensive experience with using and promoting Aquatabs. PSI's communication and behaviour change campaign will be designed with assistance from US-based Partners for Appropriate Technologies in Health (PATH). PATH is engaged in India and elsewhere on a Gates-funded learning initiative on household water treatment

Additional financial support for the trial will be provided by PATH, the United States Agency for International Development, Medentech, Ltd. and the American Chemistry Council. Neither PSI nor any of the other financial supports for the trial will play any role in the collection or analysis or the data or in the preparation of the reports or papers presenting the results of the study.

4.2. Staffing

Thomas F. Clasen, JD, PhD, a Senior Lecturer at the London School of Hygiene and Tropical Medicine and the Mailman School of Public Health, Columbia University, will be the principal investigator for the Study. Dr. Clasen is a leader in research around household water treatment in emergency and development settings. He has conducted trials has conducted household-based studies assessing the microbiological assessment and/or health impact of water quality interventions in the Congo, Ethiopia, Vietnam, Peru, Bolivia, the Dominican Republic, Colombia, Bangladesh and India. He will assist in study design, supervision, analysis and reporting. Sophie Boisson, a research Fellow at LSHTM and an epidemiologist with field experience in the Congo, Ethiopia, India, Peru, and the Dominican Republic will manage the study on-site. Dr LP Singh and Dr Vinod Kumar from the Indian Institute for Health Management Research (IIHMR) will be responsible for the process evaluation and cost-effectiveness components of the study and will provide overall support to the design, data collection and analysis. The researchers will be joined by one or more research fellows, doctoral students, MSc students at LSHTM and IIHMR.

5. References

Austin CJ (1992). Investigation of in-house water chlorination and its effectiveness for rural areas of the Gambia [dissertation]. Tulane University School of Public Health and Tropical Medicine, New Orleans (LA)

Clasen T, Edmondson P (2006). Sodium dichloroisocyanurate (NaDCC) tablets as an alternative to sodium hypochlorite for the routine treatment of drinking water at the household level. *Int'l J. Hyg. & Environ. Health* 209:173-181

Clasen T, Haller L, Walker D, Bartram J, Cairncross S (2007). Cost-effectiveness analysis of water quality interventions for preventing diarrhoeal disease in developing countries. *J. Water & Health* 5(4):599-608

Clasen T, Roberts I, Rabie T, Schmidt W-P, Cairncross S (2006). Interventions to improve water quality for preventing diarrhoea (Cochrane Review). In: The Cochrane Library, Issue 3, 2006. Oxford: Update Software

Clasen T, Saeed T, Boisson S, Edmondson P, Shipin O (2007). Household-based chlorination of drinking water using sodium dichloroisocyanurate (NaDCC) tablets: a randomized, controlled trial to assess microbiological effectiveness in Bangladesh. *Am J. Trop. Med. & Hyg.* 76(1):187-92

Cogill Bruce (2003). *Anthropometric Indicators Measurement Guide*.Food and Nutrition Technical Assistance Project, Academy for Educational Development, Washington, D.C.

Fewtrell L, Kaufmann R, Kay D, Enanoria W, Haller L , Colford J (2005). Water, sanitation, and hygiene interventions to reduce diarrhoea in less developed countries: a systematic review and meta-analysis. *Lancet Infect Dis* 5: 42-52

Hunter PR. Household Water Treatment in Developing Countries: Comparing Different Intervention Types Using Meta-Regression. *Environ Sci Technol* 2009.

Jain S, Sahanoon OK, Blanton E, Schmitz A, Wannemuehler KA, Hoekstra RM, et al. Sodium dichloroisocyanurate tablets for routine treatment of household drinking water in periurban Ghana: a randomized controlled trial. *Am J Trop Med Hyg* 2010;82(1):16-22.

Kirchhoff LV, McClelland KE, Do Carmo Pinho M, Araujo JG, De Sousa MA, Guerrant RL (1985). Feasibility and efficacy of in-home caer chlorination in rural North-eastern Brazil. *J. Hyg(Lond).*94(2): 173-80

Mausezahl D, Christen A, Pacheco GD, Tellez FA, Iriarte M, Zapata ME, et al. Solar drinking water disinfection (SODIS) to reduce childhood diarrhoea in rural Bolivia: a cluster-randomized, controlled trial. *PLoS Med* 2009;6(8):e1000125.

Morris SS, Cousens SN, Kirkwood BR, Arthur P, Ross DA. Is prevalence of diarrhea a better predictor of subsequent mortality and weight gain than diarrhea incidence? *Am J Epidemiol* 1996;144(6):582-8.

Parashar UD, Bresee, JS, Glass, RI (2003). The global burden of diarrhoeal disease in children. *Bull. WHO* 81(4): 236.

RADWQ (2008). Summary of results of rapid drinking water quality analysis. Geneva: World Health Organization (in preparation)

Roberts L, Chartier Y, Chartier O, Malenga G, Toole M, Rodka H. Keeping clean water clean in a Malawi refugee camp: a randomized intervention trial. *Bull World Health Organ* 2001;79(4):280-7.

Schmidt WP, Boisson S, Genser B, Barreto M, Baisley K, Filteau S, et al. Weight-for-age z-score as a proxy marker for diarrhoea in epidemiological studies. *J Epidemiol Community Health* 2009.

UNICEF (2009). Diarrhoea: why children are still dying and what can be done. New York: United Nations Childrens Fund

WHO/UNICEF (2000). Global Water Supply and Sanitation Assessment. Geneva: The World Health Organization and the United Nations Children's Fund.

WHO (2002). The World Health Report 2002. Geneva: The World Health Organization. Available at http://www.who.int/whr/2002/en/

WHO (2004). The World Health Report 2004. Geneva: The World Health Organization. Available at http://www.who.int/whr/2004/en/

WHO (2005). The world health report 2005: Make every mother and child count. Geneva, World Health Organization (http://www.who.int/whr/2005/en/).

WHO (2007). Combating waterborne disease at the household level. Geneva, World Health Organization

WHO (2008). Drinking Water Quality Guidelines, Vol. 1, Third Edition, Second Addendum. Geneva: World Health Organization

WHO/UNICEF, 2004. Meeting the MDG drinking water and sanitation target: a mid-term assessment of progress. Geneva: World Health Organization and UNICEF

WHO/UNICEF (2008). Progress in Drinking-water and Sanitation: special focus on sanitation. Geneva: World Health Organization and United Nations Children's Fund Joint Monitoring Program

Wright J, Gundry S, Conroy R (2004). Household drinking water in developing countries: a systematic review of microbiological contamination between source and point of use. *Trop Med Intl Health* 9(1): 106-117
